# *PAX5* alterations in B-cell acute lymphoblastic leukemia

**DOI:** 10.3389/fonc.2022.1023606

**Published:** 2022-10-25

**Authors:** Zhilian Jia, Zhaohui Gu

**Affiliations:** ^1^ Department of Computational and Quantitative Medicine, Beckman Research Institute of City of Hope, Duarte, CA, United States; ^2^ Department of Systems Biology, Beckman Research Institute of City of Hope, Duarte, CA, United States

**Keywords:** *PAX5*, *PAX5* alterations, B cell development, B-cell acute lymphoblastic leukemia, driver genetic lesions, B-ALL subtype, PAX5alt, *PAX5* P80R

## Abstract

PAX5, a master regulator of B cell development and maintenance, is one of the most common targets of genetic alterations in B-cell acute lymphoblastic leukemia (B-ALL). *PAX5* alterations consist of copy number variations (whole gene, partial, or intragenic), translocations, and point mutations, with distinct distribution across B-ALL subtypes. The multifaceted functional impacts such as haploinsufficiency and gain-of-function of PAX5 depending on specific variants have been described, thereby the connection between the blockage of B cell development and the malignant transformation of normal B cells has been established. In this review, we provide the recent advances in understanding the function of PAX5 in orchestrating the development of both normal and malignant B cells over the past decade, with a focus on the *PAX5* alterations shown as the initiating or driver events in B-ALL. Recent large-scale genomic analyses of B-ALL have identified multiple novel subtypes driven by *PAX5* genetic lesions, such as the one defined by a distinct gene expression profile and *PAX5* P80R mutation, which is an exemplar leukemia entity driven by a missense mutation. Although altered *PAX5* is shared as a driver in B-ALL, disparate disease phenotypes and clinical outcomes among the patients indicate further heterogeneity of the underlying mechanisms and disturbed gene regulation networks along the disease development. In-depth mechanistic studies in human B-ALL and animal models have demonstrated high penetrance of *PAX5* variants alone or concomitant with other genetic lesions in driving B-cell malignancy, indicating the altered *PAX5* and deregulated genes may serve as potential therapeutic targets in certain B-ALL cases.

## Background

B lymphocytes are known for generating countless high-affinity antibodies against foreign pathogens. The development of B cells starts in the bone marrow, where the hematopoietic stem cells hierarchically differentiate into fate-restricted progenitors, eventually giving rise to immature B cells heading to the spleen, where B cells further differentiate into mature B cells. This process is highly elaborate and orchestrated by a combination of intracellular mechanisms and external stimuli. Among them, PAX5 (paired box 5), or BSAP (B-cell-specific activator protein), is the pivotal transcription factor commanding the B cell development. It has been well-established that PAX5 is not only required to initiate B-lineage commitment but also essential for the maintenance of B cell identity by repressing signature genes of other lineages during the whole differentiation process.

Accompanied by the gradually uncovered mechanisms of PAX5 in normal B lymphopoiesis, extensive studies have revealed that deregulated PAX5 activities by somatic or germline alterations may lead to B-cell malignancies. Recent advances in genome-wide assays have greatly accelerated the discovery of genomic variants in B-cell acute lymphoblastic leukemia (B-ALL). SNP microarray and DNA sequencing of large cohorts of pediatric and adult B-ALL samples revealed diverse genetic lesions, of which *PAX5* was ranked the most frequently altered gene being detected in around one-third of B-ALL cases (**Table 1**) (1, 2, 6). The prevalence of *PAX5* alterations has been continually emphasized in different cohorts of B-ALL (3, 5, 7, 9, 10, 12, 15), with two B-ALL subtypes even defined by *PAX5* genetic lesions and distinct gene expression profiles (GEPs) (16). *PAX5* genetic lesions are heterogeneous, including deletions, rearrangements, sequence mutations, and focal intragenic amplifications (iAmp), which lead to the haploinsufficiency or gain-of-function of PAX5 depending on specific variants (1, 2, 8, 16). Rather than secondary events, increasing evidence from recent multi-omics and mechanistic studies has demonstrated that *PAX5* alterations can function as the initiating genetic lesions for B-ALL. In this review, we summarize recent advances in understanding the function of PAX5 in both normal and malignant B cells, with a focus on the *PAX5* alterations as founder events in B-ALL.

**Table 1:** *PAX5* alterations in B-ALL.

Cohort	Platform	Comments on *PAX5* alteration	Ref.
242 pediatric ALL	SNP array	*PAX5* gene is the most frequent target of somatic mutation, being altered in 31.7% of cases.	(1)
40 pediatric ALL	SNP array	Cell cycle and B cell related genes, including *PAX5*, are the most frequent mutated genes.	(2)
304 ALL samples	SNP array	Deletion of *PAX5* in 51% BCR::ABL1 cases, of which 95% have a deletion of *IKZF1*.	(3)
61 pediatric B-ALL (diagnosis & relapse)	SNP array	Around 50% of B-ALL have CNAs in genes known to regulate B-lymphoid development, especially in *PAX5* and *IKZF1* genes.	(4)
399 pediatric ALL	SNP array	7 cases harbor *PAX5* fusions.	(5)
221 pediatric B-ALL (high-risk)	SNP array, GEP array, target sequencing	*PAX5* CNA is involved in 31.7% of patients; P80R is the most frequent mutation.	(6)
466 pediatric ALL	FISH	*PAX5* rearrangements occur in 2.5% of B-ALL.	(7)
117 adult B-ALL	FISH, qPCR, target sequencing	*PAX5* is mutated in 34% of adult B-ALL. P80R is the most frequent point mutation. *PAX5* deletion is a secondary event.	(8)
153 adult and pediatric B-ALL with 9p abnormalities	SNP array, FISH	*PAX5* has internal rearrangements in 21% of the cases. Malignant cells carrying *PAX5* fusion genes displayed a simple karyotype.	(9)
89 Ph^+^ B-ALL	SNP array	*PAX5* genomic deletions were identified in 29 patients (33%). In all cases, the deletion was heterozygous.	(10)
Two B-ALL families	WES, SNP array	Germline *PAX5* G183S confers susceptibility to B-ALL.	(11)
116 B-ALL	MLPA	5 cases with *PAX5* intragenic amplifications were identified.	(12)
One B-ALL family	SNP array	A third B-ALL family carrying germline G183S mutation.	(13)
798 adult B-ALL	GEP array, SNP array, RNA-seq	38% of Ph-like B-ALL have *PAX5* alterations. Enrichment of CNA of *IKZF1*, *PAX5*, *EBF1*, and *CDKN2A/B* observed in the Ph-like subtype.	(14)
79 B-ALL with *PAX5* iAmp	MLPA, FISH, SNP array	*PAX5* iAmp defines a novel, relapse-prone subtype of B-ALL with a poor outcome.	(15)
1,988 B-ALL	RNA-seq, WGS, WES, SNP array	Detailed description of *PAX5* alterations in B-ALL. Defined the PAX5alt and PAX5 P80R subtypes.	(16)
110 pediatric B-others	RNA-seq, WES, SNP array	*PAX5* fusions, iAmp and P80R mutations are mutually exclusive, altogether accounting for 20% of the B-other group. *PAX5* P80R is associated with a specific gene expression signature.	(17)
250 B-ALL	DNA methylation array, WES, RNA-seq	16 patients with P80R grouped into an individual subgroup with biallelic *PAX5* alterations.	(18)
1,028 pediatric B-ALL	SNP array	20 cases of *PAX5* P80R with intermediate or poor outcome compared to the rest of this cohort.	(19)
One B-ALL family	WES, RNA-seq	*PAX5* R38H germline mutation was identified in a family with B-ALL.	(20)

CNA, copy number alterations; GEP, gene expression profile; WGS, whole genome sequencing; WES, whole exome sequencing; FISH, fluorescence in situ hybridization; MLPA, multiplex ligation-dependent probe amplification; RNA-seq, whole transcriptome sequencing.

## The function of PAX5 in B cell development

**Figure 1:**
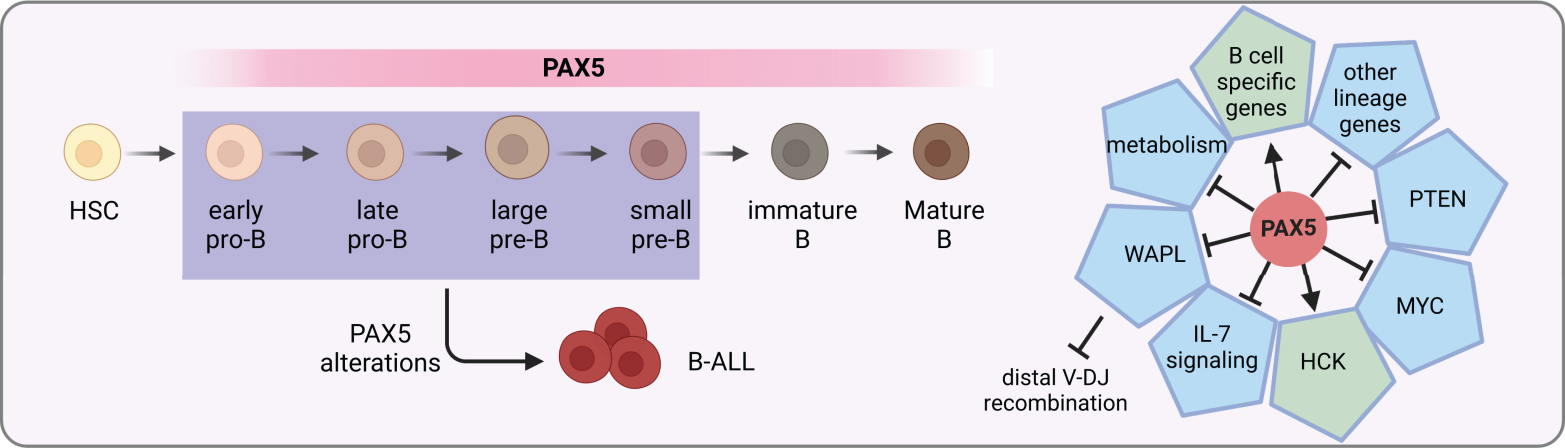
PAX5 functions in B cell development. *PAX5* is expressed during the whole B cell developmental stages. It activates the expression of B cell specific genes, while at the same time represses the expression of other lineage genes to initiate and maintain the B cell identity. In MYD88-driven B-cell lymphomas, it activates the pro-survival kinase HCK (21). In addition, it activates PI3K signaling *via* PTEN inhibition to stimulate follicular B cell proliferation (22). Furthermore, PAX5 safeguards leukemic transformation by limiting glucose and energy supply, inhibiting IL-7 signaling as well as *MYC* expression (23–25). Finally, PAX5 also has an essential role in the V(D)J recombination of the *IgH* locus by repressing *WAPL* expression (26). PAX5 alterations with compromised activity can lead to developmental arrest of B cells, which are commonly seen in B-ALL.

The multifaceted roles of PAX5 in B cell development and differentiation have been gradually unveiled. In blood cells, *PAX5* expression is exclusively restricted to the B lineage, beginning from the early pre-pro B cells and maintained through the whole process of B cell development (**Figure 1**) (27). Expression levels of *PAX5* are correlated with B cell developmental stages (28). During terminal differentiation from mature B cells to plasma cells, physiological down-regulation of *PAX5* is observed (29). This repression is not necessary for plasma cell development but essential for optimal IgG production (30). Constitutive deletion of *Pax5* in mice failed to produce mature B cells owing to a complete arrest of B lymphopoiesis at an early pro-B stage in the bone marrow (31). In contrast, B cell development is blocked at an earlier stage even before the appearance of B220^+^ progenitors in the fetal liver, suggesting different roles of PAX5 in fetal and postnatal B lymphopoiesis (31, 32). Without *Pax5*, pro-B cells retain lineage-promiscuous capacity that can differentiate into other lineages upon stimulation with proper cytokines (32–34). Therefore, PAX5 is not only required for B lymphopoiesis initiation but also continuously required for its maintenance (34). Conditional inactivated *Pax5* expression from pro-B to mature B cell stages leads to down-regulation of B-cell-specific genes and preferential loss of mature B cells, indicating that PAX5 is essential for maintaining the identity of B cells during late B lymphopoiesis (35). Further investigation of deleting *Pax5* in immature B cells in the spleen results in the loss of B-1a, marginal zone, and germinal center B cells as well as plasma cells (22). Finally, *Pax5*-deficient follicular B cells fail to proliferate due to the inhibition of PI3K signaling *via* PTEN up-regulation (22).

PAX5 safeguards the development of B cells by tailoring the gene expression profile towards the B-lineage program. On one hand, it up-regulates the expression of B-cell-specific genes such as *CD19* and *BLNK* (36). On the other hand, it down-regulates lineage-inappropriate genes such as *FLT3* and *CCL3* to suppress alternative lineage choices (**Figure 1**) (33, 37). Both activation and repression require its continuous expression (37). ChIP analysis revealed that through binding to promoters and enhancers, PAX5 directly regulates 44% of previous identified PAX5-activated genes and 24% of repressed genes (38). For PAX5 activated genes, it can induce active chromatin marks at their regulatory elements (36, 38). For example, PAX5 activates *HCK* transcription by inducing active chromatin in the *HCK* promoter in MYD88-mutated lymphoma cells (21). Conversely, it can also modify the chromatin state of its repressed genes by eliminating active histone marks (38).

PAX5 is part of a complex network of transcription factors orchestrating B cell development, including IKZF1, E2A, EBF1, and RUNX1 (23, 39, 40). Together with IKZF1, PAX5 functions as a metabolic gatekeeper to restrict glucose and energy supply. Heterozygous deletion of *Pax5* releases this restriction and increases glucose uptake and ATP levels (23). Furthermore, PAX5 is found in a physiological complex together with IKZF1 and RUNX1. Specifically, over 65% of PAX5 binding sites identified in mouse pre-B cells are overlapped with regions bound by either IKZF1, RUNX1, or both, suggesting that they are part of a regulatory network sharing a multitude of target genes (40). In addition, PAX5 and EBF1 are actively involved in a reciprocal positive regulatory loop (41, 42), yet with opposing roles in *Myc* regulation through binding to the *Myc* promoter (24). They also cooperatively regulate IL-7 signaling and folate metabolism (25).

A signature feature of B cells is the recombination of *V_H_DJ_H_* segments to generate a functional *immunoglobulin heavy chain (IgH)* gene for B cell receptor and antibody coding. PAX5 contributes to the diversity of the antibody repertoire by balancing distal-proximal *V_H_* gene choices. The first insight into the *V_H_DJ_H_* recombination role of PAX5 was provided by the observation that, in *Pax5*-deleted mouse pro-B and pre-B-I (large pre-B) cells, recombination of distal but not proximal *V_H_* genes was dramatically compromised (43, 44). Further experiments uncovered that PAX5 can mediate spatial organization of the *Igh* locus to balance the accessibility of distal and proximal *V_H_* genes (45). The mystery of this spatial regulation remained unsolved for more than a decade. Recently, it was found that PAX5 specifically inhibits the expression of *WAPL*, which encodes an architectural protein that releases the cohesin complex from chromatin (**Figure 1**) (26). With decreased levels of WAPL protein, chromatin loops are able to extrude for a longer distance to spatially connect the distal *V_H_* genes with the recombination center during the loop extrusion process (26). Thus, PAX5 fulfills a master regulator role for B lymphopoiesis by, but not limited to, inducing B-lineage commitment, maintaining B cell identity, and regulating *V_H_DJ_H_* recombination.

## Copy-number alterations of *PAX5* in B-ALL

Deletion is the most frequent form of copy number alteration of *PAX5* in B-ALL (1, 6, 8, 16). *PAX5* deletions usually affect only one allele, with either no expression or expression of truncated proteins lacking functional domains, resulting in loss of function of this allele (1). These monoallelic *PAX5* deletions in B-ALL are observed on different scales, from as focal as deletion of exons within *PAX5* gene body, to as large as loss of 9p arm or whole chromosome 9 where *PAX5* gene is located (1). In B-ALL, *PAX5* deletions are commonly concurrent with complete loss of *CDKN2A/B* genes, which encode key cell cycle regulators also situated in chromosome 9p (8, 46). Notably, *PAX5* deletions are associated with complex karyotype which is thought to be a secondary or late event, indicating the requirement of other oncogenic lesions to cause overt malignant transformation (1, 8). In support of this notion, *PAX5* deletions were found in over 50% of BCR::ABL1 and 18% of TCF3::PBX1 B-ALL cases (3, 8, 10), and were enriched in Ph-like B-ALL patients as well (14).

Studies using mouse models showed that haploinsufficiency of *Pax5* caused by monoallelic deletion exerted susceptibility of B cell transformation. Mice with heterozygous loss of *Pax5* show normal B cell development and never develop leukemia (47). But with the acquisition of other oncogenic events, they can spontaneously develop B-lineage leukemia. For example, *Pax5^+/-^* cooperated with STAT5 activation can initiate B-ALL with full penetrance in mice (47). Furthermore, compound heterozygous mutations in *Pax5* and *Ebf1* dramatically increase ALL frequency in mice (48), which is associated with the hyperactivation of the IL-7 signaling pathway (25). When synergized with *BCR::ABL1* in hematopoietic stem cells, *Pax5^+/-^* gives rise to B-ALL with shorter latencies and high incidence compared to *BCR::ABL1* alone (49). This synergistic effect may explain the frequent *PAX5* deletions in BCR::ABL1 B-ALL cases (3, 10). Finally, in *Pax5^+/-^* mice, *Jak3* mutations following postnatal infections can also act as a secondary hit for leukemic transformation (50).

The indispensable role of *PAX5* in B-lineage maintenance also explains the monoallelic but not biallelic deletions of *PAX5* observed in B-ALL cases. Complete loss of *Pax5* in mice resulted in the lack of B cells, growth retardation, and premature death (31). When *Pax5* deficiency is restricted to mature B cells to circumvent premature death, these cells dedifferentiate back into uncommitted progenitors and develop aggressive progenitor cell tumors instead of B-ALL (51). Therefore, *PAX5* deletions often disrupt only one allele and act as cooperating events in B-ALL.

## Translocations

**Figure 2:**
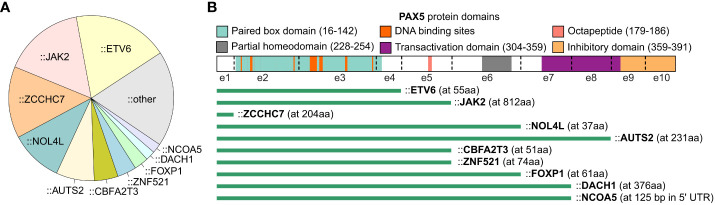
PAX5 rearrangements in B-ALL. The summary of PAX5 rearrangements is based on the result from the 1,988 B-ALL cohort (16). **(A)** Distribution of PAX5 fusion partners. The fusion partners observed in at least 2 B-ALL cases are annotated in the pie chart, and the singletons are merged into the “other” group. **(B)** Scheme of PAX5 rearrangements with recurrent partner genes. The most common isoform of each fusion is shown. The green bars indicate the remaining part of the PAX5 protein. The starting amino acid (aa) of the fusion partners are annotated in parentheses. All the rearrangements reserve the paired box DNA binding domain of PAX5, except the fusions with *ZCCHC7*, a proximal gene commonly fused with *PAX5* by focal deletion.

Translocations resulting in *PAX5* rearrangements occur in around 2.5% of pediatric and 1% of adult B-ALL patients (8). The majority of the rearrangements produce chimeric genes encoding proteins that retain the DNA-binding paired box domain and nuclear localization signal of PAX5, but with C-terminal domains adopted from the fusion partners (7) (**Figure 2**). A variety of partner genes, including transcription factors, structural proteins, and signal transducers, have been identified to fuse with the *PAX5* gene (7, 16).

With the intact paired domain, these fusion proteins are thought to bind DNA and act as dominant-negative proteins to interfere the wild-type (WT) PAX5 activities (1, 52–54). Notably, PAX5 protein consisting of only the paired domain cannot compete with full-length PAX5 for DNA binding *in vivo* (55), suggesting that the C terminal of PAX5 may contribute to DNA binding through unknown mechanisms. Indeed, PAX5 fusions with different partners display distinct DNA binding and gene regulation activities which should be examined case by case (56). In general, transient reporter assays revealed that PAX5 fusions functioned as a dominant-negative regulator for WT PAX5 through binding to PAX5-target sequences (5, 52). Some of the fusions, such as PAX5::C20S and PAX5::ETV6, showed stable DNA binding activity through forming oligomers due to the presence of oligomerization domains of the fusion partners (54, 56). As one exception, PAX5::PML barely showed DNA-binding activity but interfered with PAX5 regulatory activity through association with PAX5 proteins (53).

In contrast to deletions, *PAX5* fusions are commonly observed in leukemic cells displaying a relatively normal karyotype, indicating that they are founder lesions in leukemogenesis (9). In addition, *PAX5* fusions, except *PAX5*::*JAK2* and *PAX5*::*ZCCHC7*, are observed in over 30% of PAX5alt B-ALL, a recently reported subtype defined by various *PAX5* alterations and a distinct gene expression profile (16). Consistently, *PAX5* rearranged with *ETV6*, *ELN*, and *PML* were verified to be the primary oncogenic drivers in transgenic mice (55, 57, 58). PAX5::ETV6, the most recurrent PAX5 fusion in B-ALL, contains three domains that contribute to DNA binding behavior, which are the paired box and helix-loop-helix domain of PAX5, and the DNA binding domain of ETV6 (16, 56, 59, 60). PAX5::ETV6 regulates 68% of PAX5-target genes in an opposing manner when transduced into murine B cells. This opposite dominant effect might be responsible for impaired B cell development (61). When knocked-in into the mouse *Pax5* locus, PAX5::ETV6 blocked B cell development at the pro-B to pre-B transition but was insufficient to promote leukemogenesis (55). However, when crossed with *Cdkn2a/b* deletion mice, B-lineage leukemia was developed at full penetrance with frequent loss of the remaining WT *Cdkn2a/b* allele (55). Comparing to *PAX5*::*ETV6*, *PAX5*::*ELN* acts as a more potent initiating event to induce leukemia, with frequent acquisition of secondary mutations in *Ptpn11*, *Jak3*, and *Kras* genes in mice (58). Different from the transient expression *in vitro*, the PAX5 fusions expressed in murine models do not generally antagonize the WT PAX5 function but activate independent biological pathways to establish the molecular basis required for leukemic transformation (55, 58). This discrepancy may be explained by either different protein levels or distinct regulatory mechanisms between transient and *in vivo* expressed proteins (58).

*PAX5*::*JAK2* rearrangement exerts a distinct gene expression signature in B-ALL and is exclusively found in the Ph-like subtype (16, 62). It consists of the paired domain of *PAX5* and the kinase domain of *JAK2* (7). In contrast to cytoplasmatic localization of other JAK2 fusions such as BCR::JAK2 and ETV6::JAK2, PAX5::JAK2 protein is localized in nucleus and binds the PAX5 targets (62). It simultaneously deregulates PAX5-target genes while activating JAK/STAT signaling in the nucleus (62). In a constitutive knock-in mouse model, PAX5::JAK2 rapidly induced aggressive B-ALL without acquisition of other cooperating mutations (63), which unequivocally implicated that PAX5::JAK2 functions as dual hits, which are PAX5 haploinsufficiency and constitutively active kinase activity, to drive leukemogenesis (63).

There’s a rare translocation that does not produce chimeric protein but juxtaposes the *IGH* Eµ enhancer to proximity of the *PAX5* promoter, leading to dysregulation of *PAX5* expression. This translocation is found in a subset of B cell non-Hodgkin’s lymphoma cases (64, 65). When reconstructed by insertion of a *PAX5* mini gene into the mouse *Igh* locus, these mice develop aggressive T-lymphoblastic lymphomas instead of B-ALL, probably because of the expression of *PAX5* throughout the lymphoid system as a germline mutation rather than as somatic mutations in patients (66). It also reflects the potential caveats of using mouse models to mimic the B-lineage malignancies induced by PAX5 alterations (67).

## Intragenic amplification (iAmp)

*PAX5* intragenic amplifications (*PAX5*-iAmp) were reported in different B-ALL cohorts at an incidence of 0.5-1.4% (1, 15–17, 68). B-ALL cases with *PAX5*-iAmp lacked stratifying genetic markers and were mutually exclusive from other risk-stratifying alterations (12, 15). Transcriptome sequencing (RNA-seq) revealed that they formed a tight cluster in unsupervised hierarchical cluster analysis (17) and can be grouped into the PAX5alt subtype (16). Interestingly, *PAX5-*iAmp frequently harbor *CDKN2A/B* homozygous loss and trisomy 5 (15, 17). The preservation of *PAX5-*iAmp in matched diagnosis and relapse samples, as well as GEP clustering in the PAX5alt subtype, indicates that it may act as a driver lesion in B-ALL (15, 16).

**Figure 3:**
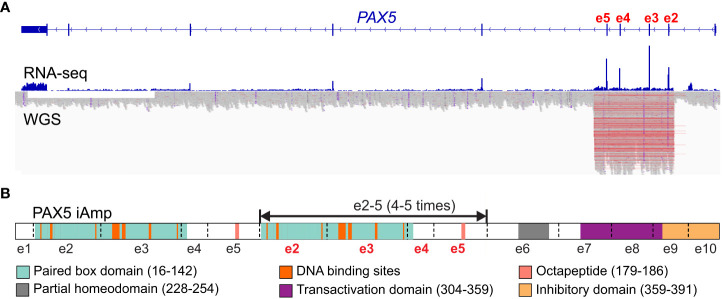
Scheme of *PAX5* intragenic amplification (*PAX5* iAmp). **(A)** *PAX5* iAmp detected by RNA-seq and whole genome sequencing (WGS (16)). The most frequently affected exons are e2-5, which are shown with increased expression levels (by RNA-seq) compared to the adjacent exons. The amplified region on the genomic level is reflected by abruptly elevated depth from WGS, which corroborates the affected exons identified from RNA-seq. **(B)** *PAX5* iAmp leads to an extended isoform of *PAX5*. Using optical genomic mapping, 4-5 extra copies of *PAX5* e2-5 were determined in the *PAX5* iAmp cases. The tandem multiplication of PAX5 paired box DNA binding domain may change its binding activity, thus altering its transcription program and disrupting B cell differentiation (68).

Whether *PAX5*-iAmp can encode structurally mutant PAX5 proteins or loss of function is still unknown. For most cases, the amplifications encompass exons 2 to 5, which encode the DNA-binding and octapeptide domains of PAX5 (15, 16, 68). Efforts have been taken to delineate the copy number of the amplified region, including chromosomal microarray analysis and multiplex ligation dependent probe amplification-based testing (15, 68). Recently, optical genomic mapping, a direct visualization method, has been applied to 3 *PAX5*-iAmp cases and found that they have an extra 4-5 copies of exons 2 to 5 inserted *in situ* in direct orientation (68) (**Figure 3**). Considering the amplified paired domain by *PAX5*-iAmp, the increased copies of the DNA-binding region may alter the binding to PAX5-target genes, thus leading to dysregulated B cell differentiation and transformation. Further functional studies are still needed to address the specific role of *PAX5*-iAmp in B-ALL.

## Alternative splicing and different isoforms

Alternative splicing of *PAX5* gene has been found during normal B cells development. By using two distinct promoters, *PAX5* can generate two different isoforms (*PAX5A* and *PAX5B*) that share the same exons 2-10 but with different exon 1 encoding N-terminal 15 or 14 amino acids, respectively (64). *Pax5A* is exclusively expressed in B cells, while *Pax5B* is active in all *Pax5*-expressing tissues such as the nervous system, testis, and B-lineage cells (64). In humans, five additional alternative isoforms have been detected in normal human B cells generated by the exclusion of exons 7, 8 and/or 9, which encode the C-terminal transactivation domain (69). The ability to induce CD19-promoter-based reporter expression by various isoforms was significantly influenced by the changes in the C-terminal domain (69). In mouse models, three additional isoforms of *Pax5* due to alternative splicing have been detected during B cells development. These isoforms arise from the exclusion of exon 2 and/or 3’ region, encoding proteins lacking part of the DNA-binding and/or the transcriptional regulatory domains, which are assumed to participate in stage-specific regulation of B cell maturation (70).

In multiple myeloma, a plasma cell disorder, diverse *PAX5* isoforms have been identified accompanied with low levels of the WT PAX5 expression (71). These noncanonical isoforms are incapable of generating functional PAX5 proteins, which may drive proliferating B cells to prematurely differentiate into plasma cells (71). In B-ALL, alternative *PAX5* isoforms missing exon 2, exons 8-9, or exon 5 have been reported (72, 73). However, considering the frequent *PAX5* intragenic deletions in B-ALL (1, 16), some of the alternative isoforms found in B-ALL might be attributed to focal deletions instead of alternative splicing.

## Point mutations

Point mutations are the second most common *PAX5* variants observed in B-ALL (7%~10%) (1, 6, 8). In 203 nonsilent *PAX5* mutations identified from 1,988 B-ALL cases (16), around three quarters are missense mutations enriched in the DNA-binding domain and are predicted to impair DNA binding by structural modelling, whereas disruptive mutations such as frameshift and nonsense are often found in the transcriptional regulatory domain (1, 16). The paired domain is a bipartite DNA-binding domain consisting of two subdomains (NTD and CTD). Each subdomain contains a helix-turn-helix motif which binds to major grooves of the DNA helix contributing to the overall binding affinity (74). Although both contribute to DNA binding, NTD determines the specificity of binding with its affinity 10 times higher than the CTD (75, 76). Coincidentally, mutations within the paired domain tend to enrich in NTD compared to CTD (52.7% to 10.3%, respectively) (16). Supporting the prevalence and importance of paired domain mutations, a study used chemical and retroviral strategies to induce random mutagenesis in *Pax5* ^+/^ *^-^* mice. For the 13 induced *Pax5* mutations acting as cooperating lesions for B-ALL, 12 are in the paired domain (67).

**Figure 4:**
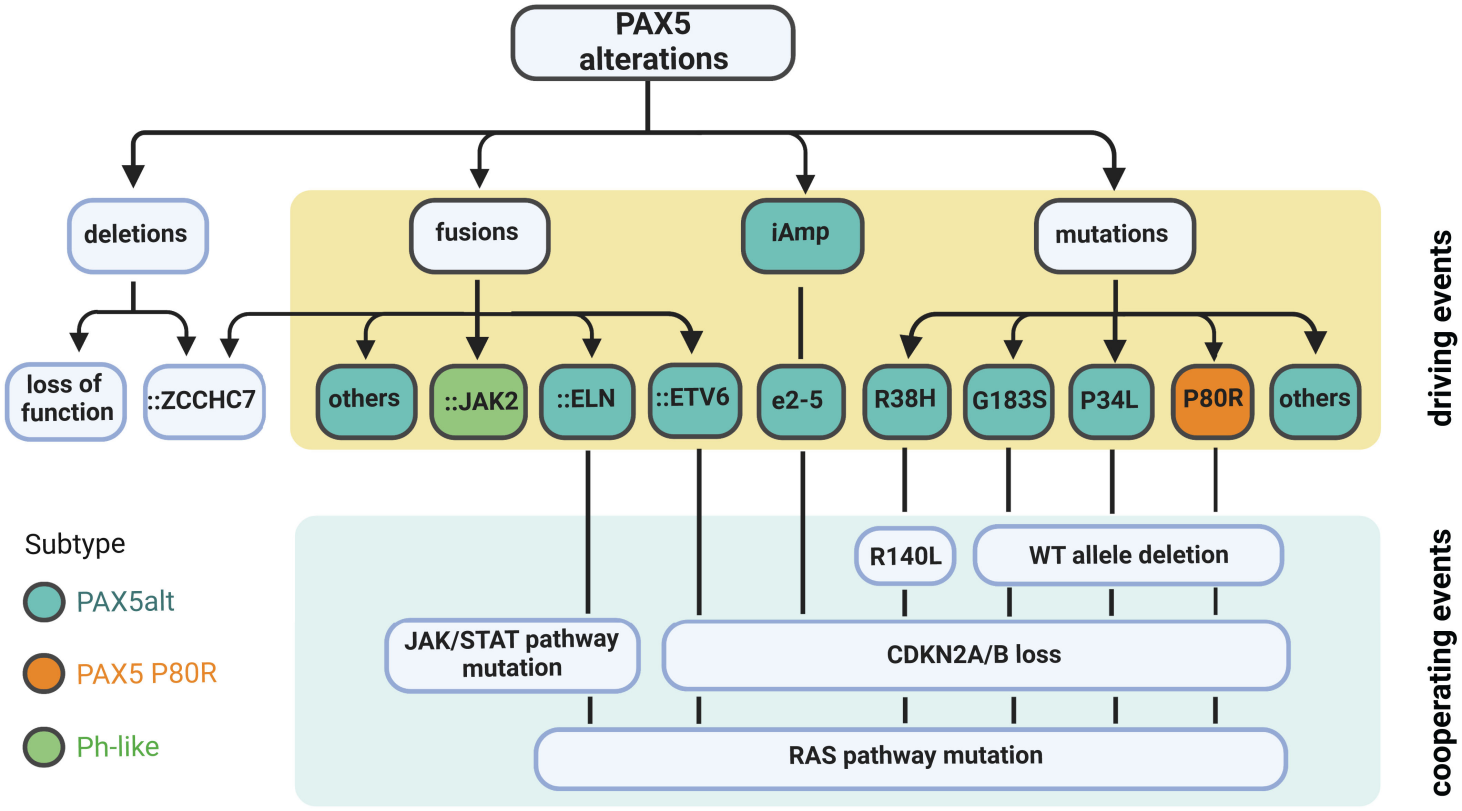
Summary of *PAX5* alterations in B-ALL. Deletion is the most common type of *PAX5* alteration in B-ALL, but generally considered as a secondary driver event. Focal deletion can lead to the concatenation of PAX5 to its adjacent gene ZCCHC7, a genetic lesion frequently observed in Ph-like and other B-ALL subtypes. *PAX5* fusions, iAmp (most commonly targeting exon 2 to 5 (e2-5)), and point mutations are highly enriched in the PAX5alt subtype (16). *PAX5* fusions and iAmp of e2-5 driven B-ALL harbor biallelic *CDKN2A/B* loss and RAS or JAK/STAT pathway mutations (16, 55, 58). *PAX5*::*JAK2*, a signature fusion of the Ph-like subtype, can act as a dual hit for B-ALL without cooperating lesions (63). *PAX5*-mutation-driven cases normally have deletion of the remaining WT allele and total loss of *CDKN2A/B*. They also frequently acquire mutations in the RAS signaling pathway. *PAX5* P80R mutation defines an independently subtype with a distinct GEP (16).

*PAX5* P80R, a substitution located in the paired domain, was identified as the most frequent sequence mutation of *PAX5* (1, 6, 8). B-ALL patients with the *PAX5* P80R mutation are classified as a novel subtype defined by this missense mutation and a highly distinct GEP (16, 77). This subtype is characterized by biallelic alterations of *PAX5*, homozygous deletion of *CDKN2A/B*, and hotspot activating mutations of RAS signaling (**Figure 4**) (16, 18). The biallelic alterations of *PAX5* are achieved by deletions, copy-neutral loss of heterozygosity, or deleterious mutations on the other allele of *PAX5* (16). Gene set enrichment analysis (GSEA) revealed dysregulation of B-cell-specific genes, suggesting that PAX5 P80R decreases the regulatory activity of PAX5 (16), probably through altering its DNA binding pattern (1). Consistently, PAX5 P80R blasts were arrested at the pre-pro-B stage (16), with T-cell antigen CD2 expressed in half of the patients (78). *PAX5* P80R B-ALL subtype, together with *DUX4r* and *ZNF384r* subtypes, frequently undergo monocytic switch (79). The patients of this subtype were reported with various levels of risk in different cohorts (16, 18, 19, 80). Therefore, further evaluation of the clinical significance of this novel subtype is still needed. The oncogenic role of PAX5 P80R has been demonstrated using constitutive knock-in mouse models. Both homozygous and heterozygous *Pax5* P80R transgenic mice developed B-lineage leukemia with almost complete penetrance. Analysis of mouse leukemia from the *Pax5* P80R heterozygous mice revealed the disruption of the remaining *Pax5* WT allele by deletion or frameshift mutations, which recapitulated the loss of *PAX5* WT allele in patient samples (16).

Non-silent *PAX5* mutations are observed in over 30% of the PAX5alt group compared to less than 5% of the other B-ALL cases (16). Over 40% of *PAX5* mutations within PAX5alt subtype are hemizygous due to loss of the *PAX5* WT allele. The two most recurrent mutations enriched in this subtype are R38H and R140L, which are located in the NTD and CTD of the DNA binding domain, respectively. Notably, 10 of 11 R140L mutations were found co-occurrent with R38H in the same patients (16). RNA-seq of one familial B-ALL case observed that these two mutations were detected on different alleles (20). PAX5alt cases with *PAX5* mutations (except R38H and R140L) are commonly observed with loss of the remaining *PAX5* WT allele and total deletion of *CDKN2A/B* genes. They are also enriched with RAS signaling pathway mutations as cooperating events (**Figure 4**) (16). Besides *PAX5* mutations, *PAX5* rearrangements and intragenic amplifications were also reported as signature genetic lesions of the PAX5alt group (16). The prognosis of this subtype is significantly worse than *PAX5* P80R (16), especially in adult cases (78). Within the PAX5alt subtype, patients with *IKZF1* deletions were observed with even worse prognosis (81). In conclusion, the large collection of *PAX5* point mutations in B-ALL with unique gene expression features implies that besides P80R, certain *PAX5* mutations may also act as initiating driver events. Further direct experimental evidence is needed to test this hypothesis.

## Germline variants and B-ALL susceptibility

*PAX5* germline variants have been identified in multiple familial B-ALL studies. Although rare, the existence of certain familial B-ALL cases provided compelling evidence that *PAX5* germline variants can induce B-ALL. The first evidence came from a heterozygous germline variant *PAX5* G183S, affecting the octapeptide domain of PAX5, found in three unrelated B-ALL kindreds with incomplete penetrance (11) (13). All affected cases exhibited chromosome 9p deletion that removed the *PAX5* WT allele and caused homozygous deletion of *CDKN2A/B* (**Figure 4**). Functional and gene expression analysis of the *PAX5* G183S mutation demonstrated that it significantly reduced transcriptional activity of PAX5 (11). The finding of *PAX5* WT allele deletion in *PAX5* G183S cases suggests that a complete disruption of WT *PAX5* is essential for B cell developmental arrest mediated by G183S (11, 13).

Further evidence came from another family with a high incidence of B-ALL affecting all three children, which harbored a *PAX5* R38H germline variant (20). This variant was inherited from one of the parents who didn’t develop leukemia, suggesting that additional lesions are required for full transformation. Consistently, all affected children gained mutations in the remaining *PAX5* WT allele. Specifically, two of them independently developed R140L mutation, which is commonly concomitant with R38H in sporadic B-ALL, while the remaining one had a *PAX5* frameshift mutation at Y371. In addition, all three children had *CDKN2A/B* homozygous loss and RAS signaling pathway mutations (**Figure 4**) (20). When transduced into murine *Pax5* ^-/-^ cells, *PAX5* R38H failed to regulate PAX5-target genes, suggesting that R38H impaired normal PAX5 function (20). Comparing to *PAX5* G183S germline variant, *PAX5* R38H is associated with an older onset, but both shared the feature of disrupting the PAX5 WT allele and *CDKN2A/B* genes (11, 13, 20). Taken together, these findings strengthen the conclusion that *PAX5* germline variants can confer strong B-ALL susceptibility and are associated with specific additional genetic lesions to initiate overt B-ALL.

## Therapeutic potential of *PAX5* alterations

As the most frequent genetic lesions in B-ALL, *PAX5* alterations have been demonstrated to impair B cell differentiation and give rise to overt leukemia with the acquisition of cooperating genetic lesions. In addition, ongoing *PAX5* deficiency is required for maintaining the lymphoblastic status of the malignant B cells *in vivo* (82). Based on these findings, strategies such as re-activating the differentiation potential of the malignant B cells to circumvent the developmental blockage may provide new therapeutic entry points. Indeed, restoring *Pax5* through Tet-Off the transgenic shPax5 in a mouse B-ALL model (driven by *Pax5* knockdown and constitutively active Stat5) enables differentiation and immunophenotypic maturation by reshaping the B cell development program, leading to durable disease remission (82). Remarkably, even brief *Pax5* restoration in B-ALL cells causes rapid cell cycle exit and disables their leukemia-initiating capacity (82). In addition, reconstitution of *PAX5* in B-ALL patient samples carrying *PAX5* deletions can restore an energy nonpermissive state, leading to energy crisis and cell death (23). Interestingly, forced expression of *PAX2* or *PAX8*, the two most closely related paralogs of *PAX5*, resulted in growth inhibition of the REH cell line, which carries a heterozygous *PAX5* A322fs frameshift mutation. These two paralogs complement the haploinsufficiency of *PAX5* in B-ALL cells by modulating PAX5-target genes and restoring B cell differentiation (83). Therefore, approaches that can by-pass the differentiation blockage resulting from PAX5 haploinsufficiency may lead to novel therapeutic approaches for this group of B-ALL, including but not limited to *PAX5* restoration and *PAX5* paralog activation.

The deregulated networks triggered by *PAX5* variants may offer other therapeutic strategies as well. For example, PAX5 deficiency can lead to upregulated metabolic genes and consequently increased glucose uptake and energy metabolism, which are essential for leukemic transformation (23, 49). Specifically, glucocorticoid receptor NR3C1, glucose-feedback sensor TXNIP, and cannabinoid receptor CNR2 were identified as central effectors of energy supply restriction in B cells. In addition, agonists against CNR2 and TXNIP synergized with glucocorticoids to exacerbate B-cell-intrinsic ATP depletion and restored the energy barrier against B-cell malignancy (23). Furthermore, *Pax5* heterozygosis can enhance the expression of inflammatory cytokine interleukin−6 (IL−6), which then promote proliferation of leukemia cells. Genetic down-regulation or pharmacologic inhibition of IL−6 is beneficial to leukemic cell clearance (84). In addition, as mentioned above, *PAX5*-variant-related leukemia is commonly associated with aberrant activation of the kinase pathways such as JAK/STAT and RAS signaling. On one hand, treatment with kinase inhibitors resulted in increased apoptosis of leukemic cells (50, 85). On the other hand, considering the requirement of converging genetic lesions into one principal pathway for leukemia initiation, pharmacological reactivation of suppressed divergent pathways may also provide a powerful barrier to leukemic transformation (86). Finally, since the majority of B-ALL subtypes are observed with distinct GEPs, the subtype-specific biomarkers may serve as targets for developing tailored therapies. Notably, the *MEGF10* gene was exclusively overexpressed in the *PAX5* P80R B-ALL subtype, which may serve as a biomarker as well as a potential therapeutic target for this subtype (16).

## Discussion

Recent genomic and transcriptomic analysis of B-ALL has largely advanced our understanding of PAX5 and its altered isoforms in regulating normal B cell development and driving malignant transformation (**Table 1**). It has been demonstrated that genetic alterations of *PAX5* in B-ALL commonly lead to a reduction in rather than a total loss of PAX5 activity. These observations suggest that a certain level of PAX5 activity is required in B-ALL to maintain B cell identity and sustain clonal expansion but is insufficient to execute normal B cell differentiation. Therefore, the remaining *PAX5* WT allele must be ablated by either deletions or deleterious mutations to achieve this haploinsufficiency threshold. The process of acquiring additional genetic lesions in *PAX5*-altered B-ALL and the underlying mechanisms are intriguing but still largely unknown. The mechanisms of V(D)J recombination, as well as class-switch recombination and somatic hypermutation in B cell development might be exploited to generate these oncogenic lesions.

In-depth investigation of the oncogenic roles of germline and somatic *PAX5* variants is still largely unavailable. Reconstruction of genetic lesions in mouse models to recapitulate the corresponding human disease is widely applied to approach this goal. However, cautions should be taken considering the potential phenotypical discrepancies between human diseases and mouse models. For example, the *Igh::Pax5* mice develop T-ALL instead of B-ALL observed in patients, which might be attributed to the germline nature of the fusion gene in mice (66). Moreover, the *Pax5*::*Jak2* mouse model generates a more aggressive leukemia through loss of the *Pax5* WT allele caused by uniparental disomy of the *Pax5*::*Jak2* allele, but the *PAX5* WT allele is normally retained in human *PAX5*::*JAK2* leukemia (63). Finally, *Pax5* G183S is insufficient for malignant transformation in a transgenic mouse model (16), although it has been found as a germline variant associated with strong susceptibility to human B-ALL (11, 13). Nonetheless, mouse models still play a critical role for investigating the function of *PAX5* variants in leukemic transformation.

In summary, genome-wide technologies have greatly refined the molecular diagnosis of B-ALL, at the same time leading to the discovery of diverse *PAX5* alterations as primary or secondary events in B cell transformation. In conjunction with the advanced understanding of PAX5 in B cell development, it will provide an objective basis for a better diagnosis and treatment of B-ALL.

## Author contributions

All authors listed have made a substantial, direct, and intellectual contribution to the work, and approved it for publication.

## Funding

This work was supported by the Research Start-Up Budget from the Beckman Research Institute of the City of Hope (to ZG), the Leukemia and Lymphoma Society Special Fellow Award LLS-3381-19 (to ZG) and NIH/NCI Pathway to Independence Award R00 CA241297 (to ZG).

## Conflict of interest

The authors declare that the research was conducted in the absence of any commercial or financial relationships that could be construed as a potential conflict of interest.

## Publisher’s note

All claims expressed in this article are solely those of the authors and do not necessarily represent those of their affiliated organizations, or those of the publisher, the editors and the reviewers. Any product that may be evaluated in this article, or claim that may be made by its manufacturer, is not guaranteed or endorsed by the publisher.
